# Editorial: Alternatives to Antimicrobial Growth Promoters and Their Impact in Gut Microbiota, Health and Disease: Volume II

**DOI:** 10.3389/fvets.2022.857583

**Published:** 2022-03-02

**Authors:** Guillermo Tellez-Isaias, Juan D. Latorre

**Affiliations:** Department of Poultry Science, University of Arkansas Agricultural Experiment Station, Fayetteville, AR, United States

**Keywords:** alternatives, antibiotics, microbiota, stress, inflammation

Following the success of the Research Topic “*Alternatives to Antimicrobial Growth Promoters and Their Impact in Gut Microbiota, Health, and Disease: Volume II*,” we received an invitation from Frontiers to work on Volume II. This second volume includes 31 scientific articles, in the text of individual chapters in this Frontiers Research Topic. The editors are grateful to all 197 authors that contributed and participated in the achievement of Volume II.

*Homo sapiens* reached the supremacy of animal species on our planet and has modified every single ecosystem known so far in a brief period. Without question, agriculture had paramount importance in this process, which eventually led to genetic modifications of domestic animals. Nevertheless, domestic animals' most critical genetic strategies have occurred during the last 60 years. Perhaps one of the most stunning examples is the modern broiler chicken. Today, a 42-g newborn chick increases its body weight by 25% (63 g) in 1 day; and 5,400% by 35 days when it reaches an average body weight of 2,273 grams. These astounding advances result from intense genetic selection, nutrition, health, and management programs.

Nevertheless, to accomplish the production goals, it is essential to maintain the integrity of the gastrointestinal tract (GIT) as the main organ responsible for the digestion and absorption of nutrients. Since feed conversion (the “money saver”) represents ~ 70% of the cost of production in poultry and livestock operations, subclinical forms of coccidiosis or necrotic enteritis in poultry are economically more devastating than short acute infections. As the growth period of broiler chickens shortens and feed efficiency continues to improve, health and nutrition programs are more demanding. Mainly because the changes that occur during the development of the intestine are microscopic, thus, generally ignored. Anything that affects gut health will be reflected in the health and productivity of the individual.

With the previous reflections in mind, it is easy to wonder if animal performance may soon meet a genetic and physiological upper limit. In recent years, the term “gut health” has become a standard in scientific literature and the animal production industries. Although the term gut health is vague, scientist agrees that “gut health” includes the ability of the GIT to conduct normal physiological processes and maintain homeostasis, allowing it to survive infections and non-infectious stressors. Without question, the gut is an extraordinary organ. Along with being in charge of water and feed absorption and digestion, the GIT is home to a rich and complex microbial community known as gut microbiota (1). The gut microbiota outnumbers somatic cells by a factor of 10, comprising ~300,000 genes compared to 23,000 in chickens (2, 3).

Moreover, because it contains more neurons than the rest of the peripheral nervous system, the enteric nervous system is known as animals' “second brain” (4). Furthermore, ~80% of the immune cells in the body are found in the gut-associated lymphoid tissue (GALT). The bursa of Fabricius, an essential lymphoid organ for B lymphocytes development and proliferation, is part of the GALT in birds (5). Interestingly, ~80% of plasma cells producing secretory immunoglobulin A (IgA), the most abundant immunoglobulin, are located in the GALT (6).

Enteroendocrine cells (EECs) are found throughout the GIT epithelium and produce several hormones that are involved in a variety of physiological functions such as secretion, absorption, digestion, and gut motility, as well as the pathogenesis of gut mucosa atrophy and cancers both inside and outside the GIT (7). Gastrin, secretin, cholecystokinin, insulin, and glucagon were the first GIT hormones described (8). As of today, over 50 gut hormones and bioactive peptides have been found, confirming that the gut is the body's largest endocrine organ, exhibiting a wide range of endocrinological, neuroendocrine, autocrine, and paracrine functions (9). The enterochromaffin cells, a subpopulation of numerous EECs, are responsible for 90% of the production of the neurotransmitter serotonin (5-hydroxytryptamine), which has a wide range of biological and multifaceted functions, including modulating mood, cognition, reward, learning, memory, reproduction, and numerous physiological processes, such as vomiting, vasodilation, gut motility, wound healing, and vasoconstriction (10). Surprisingly, the gut microbiota regulates the release of serotonin and other mood chemicals produced by EECs, such as dopamine, oxytocin, and endorphins (11–13). Numerous neurological illnesses such as schizophrenia, depression, Alzheimer's disease, Parkinson's disease, and autism have been connected to the type of microbiota that exists in the gastrointestinal tract (GIT), which has been proven through published studies (14, 15). When it comes to intuition, the classic adage “gut feelings” holds a lot of truth.

It is estimated that 90% of all diseases are caused by chronic inflammation in the intestine (16). The gut microbiota significantly impacts the host's biology, metabolism, nutrition, immunity, and neuroendocrine system (17, 18). These effects are mediated by short-chain fatty acids, gastrointestinal hormones, enteroendocrine, and immune cells (19). GIT motility is controlled by the enteric nervous system and hormonal networks, which is impaired in functional GIT diseases (20). The neuroendocrine network that connects the central nervous system, enteric nervous system, intestinal microbiota, and the GALT has a significant impact on the fragile intestinal epithelial barrier (21, 22). The balance of tolerance and immunity to non-self-antigens is regulated by this barrier, which comprises a single layer of enterocytes with tight intercellular junctions (23). Thus, the integrity of the gut is critical in maintaining a healthy balance between health and disease. Chronic stress, and chronic intestinal inflammation, divert significant biological resources away from development and reproduction to maintain the system in survival mode. Perhaps, a more comprehensive definition of “gut health” must include the harmonic interaction of what is known as the microbiota-brain-gut axis [(17, 24); Chalvon-Demersay et al.].

All biological and physiological processes balance the different microbiomes that live on mucosal surfaces (25). Loss of balance of the GIT microbiota (dysbiosis) in the GIT leads to loss of intestinal integrity (26). Microbes in the small intestine are affected by the ingredients of the diet and the viscosity of gut contents (27).

To meet their health and productivity goals, animal producers that have eliminated antibiotics from their production systems may utilize a combination of alternative products, enhanced management methods, rigorous biosecurity, and successful immunization programs. The absence of *Mycoplasma* spp. and *Salmonella* spp. from the genetic lines and the quality of the dietary items are all critical. Stress and inflammation are conditions that induce oxidative stress and lipid peroxidation of vital cellular components such as the cell and mitochondrial membranes. Damage to those organelles compromises cell homeostasis, health, and productivity.

Chronic stress and persistent inflammation are detrimental to modern animal production operations. Any cause of chronic stress, regardless of its origin (biological, physical, chemical, toxic, or psychological), will induce oxidative stress and, if persisted, chronic intestinal and systemic inflammation (28–30).

Researchers might use enteric inflammation models to examine alternative antibiotic growth promoters (AGP) and dietary supplements for livestock including poultry. Hence, our laboratory has developed several intestinal inflammatory models that include nutritional (27), management (31), chemicals (32, 33), and environmental (34). In all these models, a non-terminal approach such as serum fluorescein isothiocyanate-dextran concentration can assess intestinal permeability and is in good agreement with the measurement of bacterial translocation in the liver (35). Other trustworthy serum biomarkers, such as antioxidant biomarkers, have been included in our investigations, such as isoprostane 8-iso-PGF2α and prostaglandin GF2α (36); Griess, superoxide dismutase activity, thiobarbituric acid reactive substances, and total antioxidant capacity; enterocyte biomarkers: peptide YY, enterocellular signal-regulated kinase, citrulline, and mucin 2; and Immune biomarkers: Interferon-gamma and total or specific secretory IgA (37, 38). Other biomarkers' gene expression, such as α1-acid glycoprotein; fatty acid-binding protein; interleukins (IL-8, IL-1β,); mucin 2; transforming growth factor; and tumor necrosis factor have also been shown promising results [(39); Mullenix et al.].

Our laboratory has evaluated natural alternatives to AGP for the last 20 years. While there is no “magic bullet” for preventing chronic stress-related illnesses, the Poultry Health Laboratory of the University of Arkansas has assessed alternatives to AGP. Published laboratory and field trial studies suggest that probiotics (40), direct-fed microbials (41), prebiotics (42), organic acids (43), plant extracts (44), essential oils (45, 46), and trace minerals (47), can assist in the improvement of intestinal microbial balance, metabolism, and gut integrity.

Several phytogenics have been evaluated as feed additives in animal production for nutritional purposes. However, phytogenics play an essential role in preventing several diseases in poultry due to their antioxidant, anti-inflammatory, antibacterial, antiviral, antifungal, immunomodulatory, and barrier integrity-enhancing properties. Our studies with curcumin, the principal curcuminoid of turmeric (*Curcuma longa*), a member of the ginger family (Zingiberaceae), have shown that this unique polyphenol can reduce the severity of necrotic enteritis (43); salmonellosis (44, 48); and aflatoxicosis (49) in broiler chickens; as well as coccidiosis in Leghorn chickens (36).

Over a century ago, the father of innate immunity and novel prize winner Eli Metchnikoff proposed the revolutionary idea to consume viable bacteria to promote health by modulating the intestinal microbiota (50, 51). Bacterial antibiotic resistance (“superbugs”) is a severe problem in medicine and agriculture worldwide. This concept has never been more relevant, as increasing numbers of antibiotic-resistant strains of bacteria are posing a hazard to animal and human health, with resistance mechanisms having been identified and documented for all known antimicrobials currently accessible for clinical use in the past few decades (Björkman. et al.). Due to the rise and spread of many antibiotic-resistant zoonotic bacterial infections, there is an increased public and scientific interest in the administration of therapeutic and subtherapeutic antimicrobials to animals at present. As a result of social pressures, restrictions limiting antibiotic usage in poultry and livestock operations have been established. A high-efficient food animal production system necessitates evaluating potential antibiotic alternatives to improve disease resistance. Nutritional approaches to counteracting the debilitating effects of stress, illness, and chronic inflammation may prove to be effective alternatives to antibiotics in some cases, according to recent studies (Sylte et al.; Takano et al.; Mullenix et al.; Chalvon-Demersay et al.).

Improvement of disease resistance in animals raised without antibiotics has been demonstrated to be helpful not only to the health, welfare, and productivity of the animals but also to be a key strategy in improving the microbiological safety of animal products. Recent international legislations and increasing consumer demands to withdraw growth-promoting antibiotics and limit the therapeutic use of available antimicrobials has resulted in the research and development of alternative feed additives that are presented in this Research Topic, such as:

1. Probiotics improved overall performance, intestinal epithelial mucosal integrity, immune-related cytokines, and intestinal microbiota regulation (Li Y. et al.; Amoah et al.; Wang B. et al.; Zhang Y. et al.; Sobrino et al.); decreased virulent *E. coli* colonization (Arreguin-Nava et al.); diminish parasite survival and coccidiosis (Wickramasuriya et al.); lower *Salmonella* Enteritidis colonization (Adhikari, Hernandez-Patlan et al.).
2. Prebiotics positively impacts the integrity and performance of the gastrointestinal tract (Praxedes-Campagnoni et al.); may be able to partially preserve the intestinal health of broilers from persistent exposure to aflatoxin B1 (Hernández-Ramírez et al.); improve breast muscle yield (Zhao et al.); improve rumen microbial fermentation immune function, and performance (Chen et al.) in lambs. The inclusion of Spirulina has been shown to reduce systemic inflammation- and bacterial translocation-induced in poultry (Mullenix et al.).
3. Enzymes improve weaned piglet growth performance by enhancing dietary nitrogen digestibility and inhibiting protein fermentation in the hindgut (Li H. et al.); lower intestinal lesion scores due to necrotic enteritis and increase performance in chickens (Nusairat and Wang).
4. Short and medium-chain fatty acids have also shown benefits against necrotic enteritis by improving microbial homeostasis in chickens (Gomez-Osorio et al.).
5. Phytochemicals have antibacterial activity against Gram-negative enteropathogens (Anderson et al.); reduce the inflammation and dysbacteriosis induced by *Salmonella* Typhimurium in mice (Wang B. et al.); and improve performance, intestinal health, and resistance to coccidiosis in commercial poultry (Park et al.).
6. Vitamins. During high-concentrate feeding, dietary thiamine improves rumen epithelial barrier function *via* modulating Nrf2–NFB signaling pathways in goats (Ma et al.).
7. Functional amino acids improve overall gut health in pigs and chickens (Chalvon-Demersay et al.). Antiviral activity of 5-Aminolevulinic acid has shown to have antiviral activity in Feline Coronavirus infections (Takano et al.), which can be of relevance, considering the increasing number of cases of animals naturally infected with SARS-CoV-2, especially companion animals, offering treatment to these animals in the current COVID-19 pandemic (de Morais et al.).
8. Vaccines and immunoglobulins. The Mucosal Subunit Vaccine enhances colostrum IgA and serum IgG in sows and controls enterotoxigenic *Escherichia coli* in newborn and weanling piglets (Jabif et al.). A practical and natural resource against severe *Eimeria tenella* infection, egg yolk polyclonal IgYs can gradually reduce or eliminate anticoccidials from the diet (Juárez-Estrada et al.).
9. Bile acids. *Campylobacter jejuni* colonization ability in the intestine of laying hens may be hampered by microbiota and bile acid metabolism (Asakura et al.).
10. Changes in dietary energy levels have been shown to improve performance in donkeys by manipulating microbiome and metabolome (Zhang C. et al.).
11. Evaluation of microbiome in the litter of commercial turkeys has shown promising results in the health and diseases of turkeys (Adhikari, Tellez-Isaias et al.).
12. Novel selection of *Bacillus* strains direct-fed microbials based on quantitative enzyme determination and data analysis to assess the impacts of combinations to avoid antagonistic interactions that could limit treatment efficacy (Hernandez-Patlan et al.).

Food safety concerns and the rapid and unique use of novel research methodologies derived from systems biology ('omics) and biomarkers to accurately assess “gut health” will influence the field in the future years.

## Author Contributions

GT-I: writing—original draft preparation, formatting, and editing. JL: review and design of cover. Both authors have read and approved the submitted version.

## Funding

This study was supported by USDA-NIFA Sustainable Agriculture Systems, Grant No. 2019-69012-29905. Title of Project: Empowering U.S. Broiler Production for Transformation and Sustainability USDA-NIFA (Sustainable Agriculture Systems): No. 2019-69012-29905.

## Conflict of Interest

The authors declare that the research was conducted in the absence of any commercial or financial relationships that could be construed as a potential conflict of interest.

## Publisher's Note

All claims expressed in this article are solely those of the authors and do not necessarily represent those of their affiliated organizations, or those of the publisher, the editors and the reviewers. Any product that may be evaluated in this article, or claim that may be made by its manufacturer, is not guaranteed or endorsed by the publisher.
